# Antioxidant Activity, Phytochemical Characterization, and In Vitro Antitumor Effects of Foam‐Mat Dried Mango (*Mangifera indica* L.) Pulp

**DOI:** 10.1111/1750-3841.71024

**Published:** 2026-04-14

**Authors:** Lia Igel Sodré, Rosana Bizon Carias, Priscila Borchio, Pedro Paulo Saldanha Coimbra, Ananda da Silva Antonio, Henrique Marcelo Gualberto Pereira, Maria Eduarda Flores, Carlos Eduardo Faria, Jorge da Silva Pinho, Vanessa Naciuk Castelo‐Branco, Francine Albernaz Lobo, Valdir Florêncio da Veiga‐Junior, Anderson Junger Teodoro

**Affiliations:** ^1^ Postgraduate Program in Nutritional Sciences Federal Fluminense University Niterói RJ Brazil; ^2^ Laboratory of Regenerative Medicine Arthur Sá Earp Neto University Center/Faculty of Medicine of Petrópolis (UNIFASE/FMP) Petrópolis RJ Brazil; ^3^ Laboratory of Environmental Mutagenicity Department of Biophysics and Biometry State University of Rio de Janeiro RJ Brazil; ^4^ Laboratory for Technological Development Support Institute of Chemistry (LADETEC) Federal University of Rio de Janeiro Rio de Janeiro Brazil; ^5^ Integrated Center for Food and Nutrition (CIAN) Federal Fluminense University Niterói RJ Brazil; ^6^ Food Biotechnology Laboratory (LABIOTEC), Faculty of Pharmacy Federal Fluminense University Niterói RJ Brazil; ^7^ Department of Food Science Federal University of the State of Rio de Janeiro Rio de Janeiro Brazil; ^8^ Department of Chemical Engineering Military Engineering Institute (IME) Rio de Janeiro Brazil

## Abstract

This study investigated the antioxidant, antiproliferative, and antitumor effects of dried mango pulp (DMP) on human colorectal adenocarcinoma (Caco‐2) and human osteosarcoma (MG63) cell lines. The mango pulp was dehydrated using the foam‐mat drying method, and its bioactive compounds were identified by HPLC, UHPLC–MS, and the Folin–Ciocalteu assay, in addition to an in vitro evaluation of antioxidant capacity. DMP significantly decreased cell viability in a dose‐ and time‐dependent manner, with maximal inhibition at 5 mg/mL after 96 h (78.3% for Caco‐2; 72.69% for MG63; *p* < 0.01–0.001). In addition, DMP increased apoptosis rates (*p* < 0.05) and reduced colony formation (*p* < 0.05) of both cell lines. These results suggest that DMP is a rich source of phenolic and carotenoid compounds with remarkable antioxidant activity and antitumor potential, possibly mediated by apoptosis pathways.

**Practical Applications:**

Dried mango pulp contains natural compounds with strong antioxidant activity and the ability to slow cell growth and promote cell death in laboratory models of colorectal and bone cancer. These findings suggest it could be explored in the future as a supportive ingredient in health‐focused foods or supplements.

## 1 Introduction

Cancer repreents a critical public health problem, responsible for approximately one in six deaths worldwide, with an estimated 32.6 million new cases in 2050 (Bray et al. 2024; Ferlay et al. 2024). Factors such as population aging, population growth, and the prevalence of unhealthy habits, including exposure to tobacco smoke, alcohol consumption, high body mass index (BMI), physical inactivity, and inadequate diets, are associated with an increase in cancer cases (Zhang et al. 2020; Wu et al. 2018).

In the pathophysiological context of cancer, diet plays a crucial role in its management and prevention, effectively providing a better prognosis through the consumption of fruits, vegetables, spices, and herbs, with high nutritional value, with satisfactory concentrations of fiber, vitamin C, and bioactive compounds (Hurtado‐Barroso et al. 2020).

In the broad spectrum of horticultural species consumed in this dietary perspective, *Mangifera indica* L., popularly known as mango, from the Anacardiaceae family, may be more functionally targeted in the cancer scenario, due to its content of vitamins, fibers, and phytochemicals, including phenolic compounds and carotenoids, which have well‐established biological potential in the literature (Kim et al. 2021; Lebaka et al. 2021). Mango extracts and their isolated bioactive compounds, such as polyphenols and carotenoids, have exhibited antitumor activity in in vitro studies and in animal models for several types of cancer, including breast, ovarian, colorectal, lung, and prostate adenocarcinoma (Yap et al. 2021; Mirza et al. 2021; Kim et al. 2021).

Polyphenols and carotenoids, the main bioactive compounds in mango, have antioxidant and anticarcinogenic properties, modulating essential biochemical processes in the context of cancer (Delgado‐Gonzalez et al. 2023; Esmeeta et al. 2022). Polyphenols influence cell proliferation, apoptosis, inflammation, and metastasis, in addition to affecting redox balance and the cell cycle, suppressing antiapoptotic proteins and modulating inflammation (Niedzwiecki et al. 2016). While carotenoids, fat‐soluble pigments present in abundance in mango, inhibit cell growth and induce apoptosis, interfering with the cell cycle phases and metastasis stages, such as cell adhesion, invasion, and migration. Furthermore, some studies demonstrate the efficacy of carotenoids in models of prostate, breast, lung, and colorectal adenocarcinoma cancer (Rowles and Erdman 2020; Koklesova et al. 2020).

Among the commercially relevant cultivars of *M. indica* L., cv. Tommy Atkins is widely grown and extensively used by the processing industry in major producing regions, giving practical and industrial significance to technological research (Burton‐Freeman et al. 2017). Despite its large‐scale production and market importance, mango is highly perishable, containing approximately 80% moisture and having a relatively short post‐harvest shelf life, which increases its susceptibility to enzymatic degradation and microbial spoilage (Mujumdar 2014; Bell 2007). These characteristics present logistical and economic challenges for large‐scale commercialization and export, particularly for cultivars intended for industrial processing. In this context, dehydration technologies offer an effective strategy to extend shelf life, reduce post‐harvest losses, and improve product stability while preserving phytochemical integrity (Reis et al. 2021; Lobo et al. 2017).

Regarding drying technologies, foam‐mat drying (FMD) has emerged as a promising, efficient, and scalable industrial process. This technique enables rapid moisture removal under controlled thermal conditions, potentially minimizing the degradation of thermolabile bioactive compounds (Cardoso et al. 2022). Previous studies by our research group have shown that mango pulp processed by FMD exhibits favorable technological properties and higher concentrations of phenolic compounds and carotenoids compared with other dehydration methods, such as freeze‐drying (Lobo et al. 2017, 2020). However, although the compositional advantages and industrial applicability of foam‐mat dried mango pulp (DMP) have been established, its biological activity has not yet been systematically investigated. In particular, it remains unclear whether the enhanced phytochemical profile resulting from this processing technology translates into measurable antioxidant and antitumor effects in relevant cellular models.

In this context, the present study aimed to characterize the phytochemical profile of foam‐mat DMP and to evaluate its antioxidant, antiproliferative, and in vitro antitumor effects using Caco‐2 and MG63 cell models, as well as to identify the compounds potentially associated with these biological activities through liquid chromatography–mass spectrometry (LC–MS)‐based compositional analysis.

## 2 Materials and Methods

### 2.1 Pulp Samples and Dehydration Process

Mangos of the Tommy Atkins variety (*M. indica* L.) with purple‐green skin, firm texture, and light‐yellow pulp were obtained from a certified organic supplier in Petrópolis (22°30′17″ S, 43°10′56″ W), Rio de Janeiro, Brazil. Fruits were washed in running water to remove surface dirt and sanitized in chlorinated water with hypochlorite (200 ppm) for 15 min before pulp extraction. The pulp was manually separated, blended for 10 min at maximum speed (Electrolux 1000 W), and sieved through a 16‐mesh sieve. FMD was performed using carboxymethylcellulose and soy lecithin (300 mg/100 g pulp) as foaming agents. The foam was prepared with a mixer (Arno) for 10 min, spread on trays, and dried in a forced‐ventilation oven at 70°C until a porous film formed (Lobo et al. 2017). The dehydrated mango pulp was stored in light‐protected polyethylene bags at −80°C. This method was selected for its reported higher retention of phenolic compounds and antioxidant activity in mango (Lobo et al. 2017).

### 2.2 In Vitro Antioxidant Activity, Colorimetric Assay, and Phenolic Content of DMP

Two extraction systems were used for the antioxidant tests, to recover compounds with different polarities: an aqueous extract (ultrapure water) and a hydroethanolic extract (50% ethanol, v/v), both prepared at 5 mg/mL. While water favors the extraction of highly polar compounds, the hydroethanolic solution enhances the solubilization of moderately polar phenolics and certain carotenoid derivatives, allowing broader phytochemical recovery. After homogenization and centrifugation, the supernatant was collected to remove insoluble particles and prevent interference in spectrophotometric and fluorimetric analyses. The samples (aqueous and ethanolic extracts) were prepared in three different concentrations and in triplicate to be used in antioxidant activity (1,1‐diphenyl‐2‐picrylhydrazyl [DPPH], 2,2′‐azinobis(3‐ethylbenzothiazoline‐6‐sulfonic acid) [ABTS], and oxygen radical absorbance capacity [ORAC]) and total phenolic compounds (Folin–Ciocalteu) assays according to methodologies reported by Abreu et al. (2019), adapted for a 96‐well microplate format. Readings were carried out using a microplate reader/fluorimeter (SpectraMax i3x Multi‐Mode Microplate Reader, California, USA) for all methods.

#### 2.2.1 DPPH Assay

Samples were introduced to react with 225 µL of the stable DPPH radical in a methanol solution (2.4 mg%). The reduction of the DPPH radical was gauged by measuring absorbance at 515 nm after a 15‐min reaction period. A Trolox standard (6‐hydroxy‐2,5,7,8‐tetramethylchromane‐2‐carboxylic acid) was employed, and the antioxidant potential was expressed in µmol Trolox/100 g of the sample.

#### 2.2.2 ABTS Assay

Antioxidant capacity was assessed using the ABTS^+^ radical scavenging assay. The ABTS^+^ radical was generated from a 7 mM ABTS solution incubated in the dark at room temperature for 16 h and subsequently diluted with ethanol to an absorbance of 0.70 ± 0.02 at 734 nm. Samples were mixed with 280 µL of the diluted radical solution, and absorbance was recorded after 6 min. Antioxidant activity was quantified using Trolox as a standard and expressed as µmol Trolox/100 g of sample.

#### 2.2.3 Oxygen Radical Absorbance Capacity Assay

Antioxidant capacity was evaluated using the ORAC assay. Phosphate‐buffered saline (PBS, pH 7.4), fluorescein, Trolox standards, and 2,2′‐azobis(2‐amidinopropane) dihydrochloride (AAPH) were prepared for the assay. Trolox calibration curves were generated using eight concentrations ranging from 2.5 to 20 µg/mL, with PBS serving as the blank and control. Trolox standards and sample extracts were analyzed in duplicate at increasing concentrations. Fluorescein (120 µL) was added to each well, followed by 60 µL of AAPH, except in control wells. Fluorescence was monitored at 485/520 nm (excitation/emission) using an automated 96‐well microplate reader. Antioxidant capacity was calculated from the area under the fluorescence decay curve and expressed as µmol Trolox/100 g of sample.

#### 2.2.4 Total Phenolic Content (TPC) Assay

TPC was determined using the Folin–Ciocalteu method. Samples were mixed with 150 µL of 10% Folin–Ciocalteu reagent and allowed to react for 5 min, followed by the addition of 120 µL of 4% sodium carbonate solution. Quantification was performed using a gallic acid calibration curve. Absorbance was measured at 750 nm by spectrophotometry, and results were expressed as mg gallic acid equivalents per 100 g of sample.

#### 2.2.5 Colorimetric Analysis

DMP color determination was obtained using a digital colorimeter (CM‐5, Konica Minolta, Osaka, Japan). The *L* ^*^ (luminosity), *a* ^*^ (red–green component), and *b* ^*^ (yellow–blue component) values were directly obtained from the colorimeter and used to calculate chromatic hue (*h*° = *b* ^*^/*a* ^*^) and chroma (*C* ^*^ = (*a* ^2^ + *b* ^2^)^½^). The luminosity scale (*L* ^*^) ranged from 0 to 100, where 0 indicated black (or dark color) and 100 indicated white (or light color).

### 2.3 Quantitative Phenolic and Carotenoid Compounds Identification by HPLC–DAD

Phenolic and carotenoid compounds from DMP were extracted separately. Phenolic extraction followed Lobo et al. (2017) with modifications: 0.5 g DMP was extracted with 80% methanol containing 0.1% HCl (v/v), vortexed (Thermo Fisher) at 2500 rpm for 15 min, and the supernatant collected. This was repeated twice with 5 mL solvent and 5 min vortexing. Supernatants were filtered (Whatman No. 1), dried in a rotary evaporator (Fisatom‐802, 35°C), resuspended in 2.5 mL methanol, and filtered (0.45 µm PTFE).

Carotenoid extraction followed O'Sullivan et al. (2017) with adaptations: 0.1 g DMP was sequentially mixed (15 s vortex) with 500 µL ethanol, 3.0 mL acetone, 1.0 mL Milli‐Q water, and 2.0 mL hexane, rested for 5 min, and the upper phase was collected and evaporated (35°C). This was repeated twice until full color removal. The residue was resuspended in 1 mL hexane and filtered (0.45 µm PTFE).

Compounds were identified using a Shimadzu HPLC–DAD (SPDM20A). Phenolics were analyzed on a C18 column (Shim‐pack VP‐ODS, 4.6 × 250 mm, 5 µm) with 0.01% formic acid in water (A) and acetonitrile (B) under the following gradient: 0–5 min, 5% B; 5–20 min, 50% B; 20–25 min, 100% A, followed by washing/re‐equilibration. Flow: 1.0 mL/min, 25°C. Carotenoids were analyzed on a C30 column (Acclaim, 4.6 × 155 mm, 5 µm) following Rodriguez‐Amaya et al. (2004), using acetonitrile (A), methanol (B), ethyl acetate (C), and 200 mM acetic acid in Milli‐Q water (D) with gradient: 0–15 min, 85% A, 7.25% B, 7.25% C, 0.5% D; 25–30 min, 65% A/17.25% B/17.25% C/0.05% D; 35–40 min, 85% A/7.25% B/7.25% C/0.5% D. Flow: 1.5 mL/min; injection: 10 µL.

Quantification used external calibration curves from HPLC‐grade standards (Sigma‐Aldrich)—β‐carotene, astaxanthin, and zeaxanthin for carotenoids, and caffeic acid, sinapic acid, gallic acid, 5‐caffeoylquinic acid, *o*‐coumaric, *p*‐coumaric, 4‐hydroxybenzoic acid, and ferulic acid for phenolics—measured at 450 nm for carotenoids and 270, 310, 320, and 325 nm for phenolics. Results were expressed in mg/100 g dry matter.

### 2.4 Qualitative Compounds Identification by UHPLC–HRMS

Qualitative compound identification was performed following the methodology of Coimbra et al. (2023), with modifications. Dehydrated mango pulp was diluted in methanol (1 mg/mL) and analyzed by ultra‐high‐performance LC (UHPLC; Dionex Ultimate 3000, Thermo Scientific, Germany) coupled to high‐resolution MS (HRMS; Q‐Exactive, Thermo Scientific, Germany). Chromatographic separation was achieved using a Syncronis C18 column (2.1 × 50 mm, 100 Å) with a stepwise gradient from 0 to 100% solvent B (0.1% formic acid in deionized water containing 5 mM ammonium formate) against solvent A (methanol) at a flow rate of 0.4 mL/min, increasing 9% B per min.

HRMS analyses were conducted using electrospray ionization in positive and negative modes over an *m/z* range of 100–900. Data‐dependent acquisition was employed for MS^2^ fragmentation. Data processing and deisotoping were carried out using MZmine v3.9.0 (Schmid et al. 2023), as detailed in Table 1. Compound annotation was achieved by matching fragmentation spectra with the GNPS database and literature sources (Ybañez‐Julca et al. 2020), applying a mass error threshold of ≤ 10 ppm (Demarque et al. 2020). GNPS “Data Analysis” parameters included a precursor ion mass tolerance of 2.0 Da, a minimum of three matched peaks, a fragment ion mass tolerance of 0.5 Da, and a score threshold of 0.7.

**TABLE 1:** Data processing steps in MZmine 3.9 UHPLC–HRMS data.

Ionization mode	Positive	Negative
	Step 1. Mass detection	
Mass detector	Exact mass	
MS level	1	
Noise level	1.3 × 10^6^	1.8 × 10^5^
MS level	2	
Noise level	1.3 × 10^6^	1.8 × 10^5^
	Step 2. ADAP chromatogram builder	
Min. group size of scan	5	
Group intensity threshold	4.0 × 10^6^	5.0 × 10^5^
Min. highest intensity	2.5 × 10^6^	5.0 × 10^5^
*m/z* tolerance	0.0 Da or 10 ppm	
	Step 3. Deisotope	
*m/z* tolerance	0.001 Da or 10 ppm	
Retention time tolerance (%)	20	
Maximum charge	3	
Representative isotope	Most intense	
	Step 4. Alignment (join aligner)	
*m/z* tolerance	0.001 Da or 10 ppm	
Retention time tolerance (%)	20	
Weight for *m/z*	75	
Mobility weight	1.000	
	Step 5. Duplicate feature filter	
*m/z* tolerance	0.001 Da or 10 ppm	
Retention time tolerance (%)	20	

### 2.5 Influence on Cancer Cells

#### 2.5.1 DMP Extract Preparation

To evaluate the action of DMP in vitro, it was added to the Dulbecco's Modified Eagle's Medium Low Glucose culture medium (DMEM; Sigma‐Aldrich, St. Louis, MO, USA), at a concentration of 5 mg/mL, homogenized in a vortex Thermo Fisher—LP Vortex Mixer) at 2500 rpm for 10 min and filtered by a vacuum pump system, pore size 0.2 µm (Corning). To prepare the other concentrations, this solution was diluted in supplemented culture medium and adjusted according to the desired concentration.

#### 2.5.2 Cell Culture

Human colon adenocarcinoma cell line (Caco‐2) and human osteosarcoma (MG63) cells were obtained from the Rio de Janeiro Cell Bank (BCRJ) and Laboratory of Biology of Eukaryotic Cells (INMETRO, Rio de Janeiro/Brazil). Cellular authenticity tests were carried out to control and guarantee the quality of the experiments, in addition to specific microbiological tests. Analysis of the short tandem repeat (STR) marker was used as a basis for screening possible cross‐contamination with other strains. The automated device BACTECTM FX BD (Becton‐Dickinson) was used to ensure the absence of bacteria and fungi. In time, to detect the presence of *Mycoplasma*, the bioluminescence method, Lucetta 2 system (Lonza, MycoAlert PLUS Mycoplasma Detection Kit, LT07‐710) was used.

Caco‐2 and MG63 were incubated in DMEM Low Glucose (DMEM‐Low; Sigma‐Aldrich, St. Louis, MO, USA), containing 20% and 10% fetal bovine serum (FBS, Invitrogen, Carlsbad, CA, USA), respectively, and 1% of ciprofloxacin (Eurofarma, Rio de Janeiro, RJ, Brazil). The incubation was maintained in a humidified atmosphere at 37°C with 5% CO_2_. Stock cultures in flasks were grown to 70% confluence and routinely subcultured.

#### 2.5.3 Cell Viability Assay

Cells were seeded in 96‐well plates at a density of 1.5 × 10^4^ cells per well and allowed to adhere for 24 h. The culture medium was then removed, and cells were washed with PBS (GIBCO). Subsequently, cells were treated with culture medium containing the extracts at concentrations ranging from 5 to 0.078 mg/mL, with the final volume adjusted to 100 µL per well. Plates were incubated for 48, 72, or 96 h. Cell viability was evaluated using the MTT assay (4,5‐dimethyl‐2‐thiazolyl)‐2,5‐diphenyl‐2H‐tetrazolium; Sigma‐Aldrich), according to Kumar et al. (2018). Absorbance was measured at 570 nm using a spectrophotometer (EVOS AMF 5000, Thermo Scientific), and cell viability was expressed as the percentage of viable cells based on nonlinear regression analysis. A 1% Triton X‐100 solution (Sigma‐Aldrich) was used as the positive control, while cells cultured in medium without extract served as the negative control.

#### 2.5.4 Clonogenic Assay

The clonogenic assay was conducted according to the methodology described by Rafehi et al. (2011). Cells were seeded in 6‐well plates at a density of 10 cells/cm^2^ in complete culture medium. After 48 h, cells were washed with PBS and treated with medium containing dehydrated mango pulp at concentrations of 2.5 and 5.0 mg/mL. Cultures were maintained for 14 days in a humidified atmosphere at 37°C with 5% CO_2_, with treatment peaking on Day 7. Subsequently, cells were washed with PBS and fixed with 4% paraformaldehyde for 1 h at room temperature. The number of colonies was quantified using an inverted microscope (Thermo Scientific—EVOS—M5000), and independent clusters with more than 50 individual cells were considered as colonies.

#### 2.5.5 Apoptosis Assay

Caco‐2 cells were seeded in 6‐well plates at a density of 1.0 × 10^4^ cells/cm^2^ in complete culture medium. After 24 h, cells were treated with dehydrated mango pulp at concentrations of 2.5 and 5.0 mg/mL in standard culture medium for 72 h. To assess apoptosis, cells were washed with PBS and detached using 0.125% trypsin/EDTA (Sigma‐Aldrich) at room temperature. Apoptotic and necrotic cells were stained with Annexin V‐FITC and propidium iodide (PI) (BD Pharmingen, San Diego, CA, USA) according to the manufacturer's instructions. Samples were quantified by flow cytometry (FACSAria III, BD Biosciences), and data were analyzed using FlowJo software.

### 2.6 Data Analysis

For data analysis, the program GraphPad Prism version 5.0 (GraphPad software Inc., La Jolla, CA, USA) was used to calculate the mean and standard deviation, as well as statistical analysis of cytotoxicity assays, cell apoptosis and assay clonogenic, as well as the DMP characterization steps (antioxidant and HPLC analysis). One‐way ANOVA test with multiple comparisons of the Tukey test compared to the respective control group was used to evaluate the statistical significance of the data. Data with *p* < 0.05 are considered statistically significant, for a reliability level of 0.95.

## 3 Results and Discussion

### 3.1 In Vitro Antioxidant Activity, Colorimetric Assay, and Phenolic Content of DMP

Antioxidant activity is closely linked to health benefits and the prevention of chronic diseases, and its in vitro assessment can be carried out using various direct and indirect analytical methods. In this study, the antioxidant capacity of DMP was evaluated using DPPH, ABTS^+^, and ORAC assays to provide a comprehensive characterization of its activity (Table 2). Mango is widely recognized for its richness in bioactive compounds with antioxidant properties. DMP exhibited particularly high phenolic content, with levels exceeding those reported for mango pulps dehydrated by FMD (103.20–224.24 mg GAE/100 g) as described by Tran et al. (2023), and surpassing phenolic contents obtained through other dehydration techniques such as heat‐pump and freeze‐drying (Chong et al. 2013; Corrales‐Bernal et al. 2014).

**TABLE 2:** Summary of the in vitro antioxidant activity in the dried mango pulp extracts.

In vitro analysis	Aqueous extract	Ethanolic extract
TPC (mg GAE/100 g)	328.57 ± 6.8	123.78 ± 7.3
DPPH (µmol Trolox/100 g)	4839.31 ± 3.3	3706.45 ± 1.6
ABTS (µmol Trolox/100 g)	655.18 ± 0.3	510.04 ± 0.1
ORAC (µmol Trolox/100 g)	5026.26 ± 10.7	8588.47 ± 15.2

*Note*: Triplicate mean and standard deviation (±) of phenolic concentrations and antioxidant activity equivalent to 100 g of dehydrated mango pulp, under the conditions of aqueous extract and ethanolic extract.

Abbreviations: g, gram; GAE, gallic acid equivalent; mg, milligram.

In both the DPPH and ABTS^+^ assays, the aqueous extract demonstrated superior antioxidant activity, concomitant with the highest TPC observed in this extract. These findings indicate that aqueous extraction is the most efficient method for recovering phenolics from DMP, suggesting that the matrix contains a predominance of polar and consequently hydrophilic compounds (Table 2).

In the DPPH radical scavenging assay, DMP showed higher antioxidant activity than mango pulps dehydrated by other methods, including hot air–cold air, hot air vacuum–microwave, heat pump, and heat pump vacuum–microwave techniques, as described by Chong et al. (2013), with reported values ranging from 761 to 2835 µmol Trolox/100 g. The antioxidant activity of DMP measured by ORAC and ABTS^+^ assays is also consistent with previous studies. Le (2012) reported antioxidant capacities of aqueous extracts from dehydrated Tommy Atkins mangoes ranging from 3220 to 6210 µmol TE/100 g (ORAC) and from 467 to 738 µmol Trolox/100 g (ABTS^+^).

Color is also an important quality indicator in food matrices, as it may reflect the deterioration or loss of pigments and other bioactive compounds of interest during dehydration. Color parameters (*L* ^*^, *a* ^*^, and b*) are shown in Table 3, corresponding to lightness/darkness (*L* ^*^), green/red (*a* ^*^), and blue/yellow (*b* ^*^). DMP exhibited a yellow–orange color (*b* ^*^ = 47.98), characteristic of fresh mango, likely due to its carotenoid content, such as β‐carotene, which confers a yellow hue (Barreiro and Barredo 2018). A slightly brownish tone was also observed, which may be attributed to non‐enzymatic browning or sugar caramelization during heating, characteristic of the Maillard reaction. This reaction may not only alter sensory attributes but also modify the functionality of bioactive compounds (Murata 2021).

**TABLE 3:** Colorimetric characterization of dried mango pulp.

Colorimetric characterization of dried mango pulp	
*L* ^*^	59.77 ± 0.20
*a* ^*^	12.87 ± 0.08
*b* ^*^	47.98 ± 0.13
C*	49.67 ± 0.13
***h*°**	74.98 ± 0.08

### 3.2 Phenolic and Carotenoid Content in DMP

According to Table 4, DMP exhibited β‐carotene as the main bioactive compound, with a content of 11.12 mg/100 g. In addition, xanthophylls such as astaxanthin and zeaxanthin were also identified in smaller quantities, resulting in a total carotenoid content of 13.83 mg/100 g in DMP. These compounds are characterized by their antioxidant activity, which is consistent with the results obtained in the DMP antioxidant assays (Mueller and Boehm 2011).

**TABLE 4:** Phenolic and carotenoid content in dried mango pulp, expressed as mg/100 g on dry basis, by HPLC–DAD.

Carotenoid compounds	Class	Formula	Content (mg/100 g DMP)	Molecular structure
**β‐carotene**	Carotenoid (carotene)	C_40_H_56_	11.12 ± 1.19	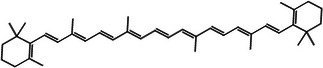
**Zeaxanthin**	Carotenoid (xanthophyll)	C_40_H_56_O_2_	1.59 ± 0.01	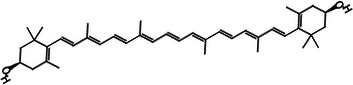
**Astaxanthin**	Carotenoid (xanthophyll)	C_40_H_52_O_4_	1.12 ± 0.00	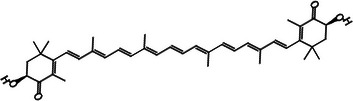
**Phenolic compounds**	**Class**	**Formula**	**Content (mg/100 g DMP)**	**Molecular structure**
**5‐caffeoylquinic acid (5‐CQA)**	Polyphenol (phenolic acid)	C_16_H_18_O_9_	4.96 ± 0.15	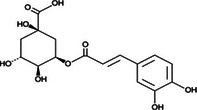
**Caffeic acid**	Polyphenol (phenolic acid)	C_9_H_8_O_4_	1.61 ± 0.00	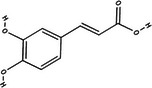
***o*‐Coumaric acid**	Polyphenol (phenolic acid)	C_9_H_8_O_3_	1.93 ± 0.34	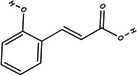
***p*‐Coumaric acid**	Polyphenol (phenolic acid)	C_9_H_8_O_3_	0.78 ± 0.03	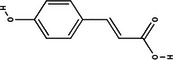
**Sinapic acid**	Polyphenol (phenolic acid)	C_11_H_12_O_5_	1.46 ± 0.00	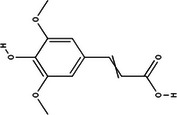
**Gallic acid**	Polyphenol (phenolic acid)	C_7_H_6_O_5_	0.90 ± 0.00	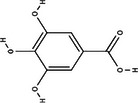
**4‐Hydroxybenzoic acid**	Polyphenol (phenolic acid)	C_7_H_6_O_3_	0.70 ± 0.08	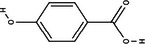
**Ferulic acid**	Polyphenol (phenolic acid)	C_10_H_10_O_4_	0.54 ± 0.02	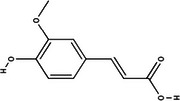

All phenolic compounds quantified belonged to the class of phenolic acids, a subgroup commonly present in mango and associated with antioxidant and anticancer activities (Esmeeta et al. 2022). Among these, ferulic, *p*‐coumaric, *o*‐coumaric, caffeic, 5‐caffeoylquinic, and sinapic acids are classified as hydroxycinnamic acids, whereas gallic acid, 4‐hydroxybenzoic acid, and chlorogenic acid (5‐caffeoylquinic acid) are benzoic acids. Chlorogenic acid was the most abundant, reaching 4.96 mg/100 g in DMP. This compound, like other phenolic acids, displays high antioxidant potential due to its aromatic phenolic ring structure, which facilitates free radical neutralization (Esmeeta et al. 2022).

Beyond quantitative analysis of DMP's bioactive profile, an untargeted chemical profiling approach was employed to further elucidate how these compounds may influence cancer cell survival and death (Table 5). A total of 22 compounds across diverse chemical classes—including polyphenols, fatty acids, carbohydrates, and lysophospholipids—were identified (Supporting Information). Although not quantified, the results confirmed DMP as a rich source of bioactive molecules, with chemical diversity consistent with the LC data (Table 5). The metabolomic profile closely aligns with previous findings (Farag et al. 2022; Lauricella et al. 2019).

**TABLE 5:** Phytochemical profile of Tommy Atkins mango pulp dehydrated by mass spectrometry (UHPLC–HRMS).

Bioactive compounds	Class	Formula	Adduct	*m/z* theoretical	*m/z* observed	Mass accuracy (ppm)	Molecular structure	Ref.
**Mangiferin**	Polyphenol (xanthone)	C_19_H_18_O_11_	[M − H]^−^	421.0776	421.0779	0.71	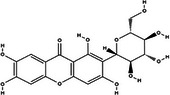	[Ybañez‐Julca et al. 2020]
**Gallic acid 4‐O‐glucoside**	Polyphenol (phenolic acid)	C_13_H_16_O_10_	[M − H]^−^	331.067	331.0672	0.06	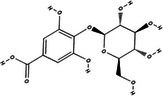	GNPS
**Quinic acid**	Cyclic polyol	C_7_H_12_O_6_	[M − H]^−^	191.0561	191.0551	−5.29	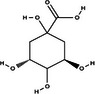	GNPS
**3‐Feruloylquinic acid**	Cyclic polyol (quinic acid)	C_17_H_20_O_9_	[M − H]^−^	367.1034	367.1047	3.54	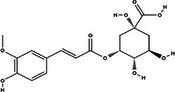	GNPS
**Citric acid**	Organic acid	C_6_H_8_O_7_	[M − H]^−^	191.02	191.0188	−6.28	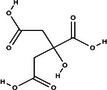	GNPS
**Palmitic acid (1‐palmitoyl‐2‐hydroxy‐sn‐glycero‐3‐phosphoethanolamine)**	Fatty acid	C_21_H_44_NO_7_P	[M − H]^−^	452.279	452.2781	−1.98	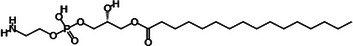	GNPS
**Linolenic acid**	Fatty acid	C_18_H_30_O_2_	[M − H]^+^	279.232	279.2317	−1.07	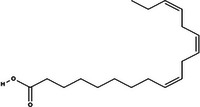	GNPS

*Note*: For mangiferin, in addition to the ion precursor, the following fragments were considered: 301.03, 421.077, 331.04, 259.02, and 271.02.

Abbreviations: *m/z*, mass‐to‐charge ratio; ppm, parts per million; Ref., References.

Mangiferin, a glycosylated xanthone characteristic of the Anacardiaceae family and *M. indica* species, was the most prominent bioactive compound identified by MS (Imran et al. 2017). Other compounds, including gallic acid, cyclic polyols, organic acids, and lipids, were also detected, corroborating the chromatographic data. The antioxidant activity observed in DMP is likely attributable to its content of bioactive compounds.

### 3.3 DMP Reduces the Viability of Colorectal Adenocarcinoma and Human Osteosarcoma Cell Lineage

**FIGURE 1:**
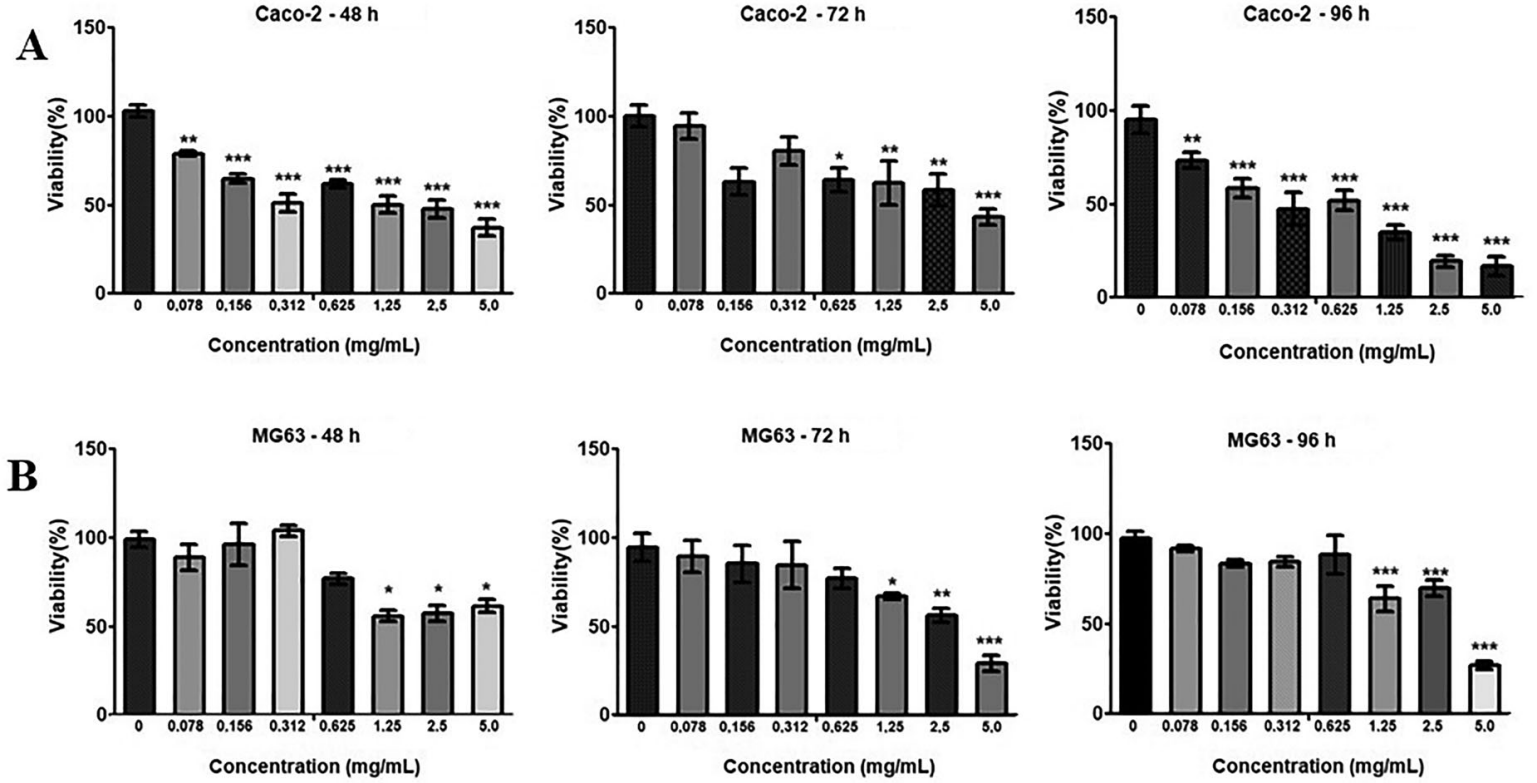
Evaluation of the effect of cell viability and cytotoxicity of dried mango pulp (PMD) after 48, 72, and 96 h of exposure in Caco‐2 (A) and MG63 (B) cell lines. Cell viability was assessed by the MTT assay. The concentrations evaluated were 5, 2.5, 1.25, 0.625, 0.312, 0.156, and 0.078 mg/mL, and the control group (cells without treatment). Results are expressed as mean (±SD). One‐way ANOVA test with multiple comparisons of the Bonferroni test compared to the respective control group (^*^ *p* < 0.05; ^**^ *p* < 0.01; ^***^ *p* < 0.001).

Several of the metabolites and bioactive compounds found in DMP (e.g., mangiferin, gallic acid, citric acid, and palmitic acid) have documented antitumoral activity in vitro and in vivo (Xiao et al. 2024; Kuang et al. 2023). To assess the effect of DMP on tumor cells, seven concentrations were tested in MG63 and Caco‐2 cells. A significant reduction in cell viability of the Caco‐2 lineage was observed in a dose‐dependent manner across all concentrations tested after 48 h of exposure (*p* < 0.01 to *p* < 0.001). After 72 h of exposure to DMP, significant reductions in cell viability were observed only at the four highest concentrations tested. After 96 h, all doses showed significant reductions in viability (*p* < 0.01 to *p* < 0.001), indicating a time‐dependent increase in DMP's effectiveness on Caco‐2 cells. This time‐dependent effect is characteristic of some bioactive compounds (Coimbra et al. 2023). At the highest concentrations of 5 mg/mL and 2.5 mg/mL, reductions in cell viability of 78.3% and 74.6%, respectively, were observed (Figure 1A).

For the MG63 cell line, DMP also induced a significant reduction in cell viability at the three highest concentrations tested (5, 2.5, and 1.25 mg/mL) with the effect becoming more pronounced over time. At the highest concentration (5 mg/mL), cell viability decreased by 69.18% after 72 h and 72.69% after 96 h of exposure to DMP (Figure 1B).

Among the compounds identified in DMP, phenolic compounds and carotenoids are notable for their in vitro antitumoral activity. Their mechanisms include induction of G2/M cell cycle arrest, modulation of autophagy, and enhancement of intracellular ROS generation (Lozano‐Casabianca et al. 2023; Vitto et al. 2022; Russo et al. 2022).

### 3.4 DMP Reduces the Number of Colonies of Caco‐2 and MG63 Cells

**FIGURE 2:**
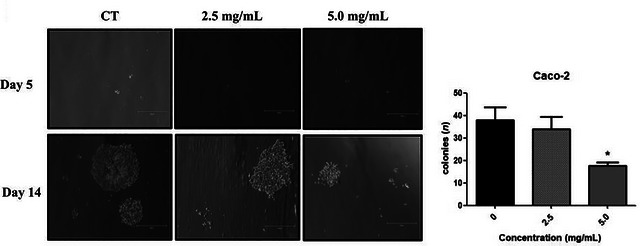
Colony formation assay with Caco‐2 cell line under treatment with dehydrated mango pulp (PMD) at concentrations of 2.5 and 5 mg/mL, compared with no treatment. Representative images are shown on the left, and quantitative analysis of the total number of colonies formed after 14 days is presented on the right. The concentration of 5 mg/mL was able to significantly inhibit colony formation when compared to the control group. One‐way ANOVA test with multiple comparisons of the Tukey test compared to the respective control group (^*^ *p* < 0.05). Scale bars = 750 µm.

**FIGURE 3:**
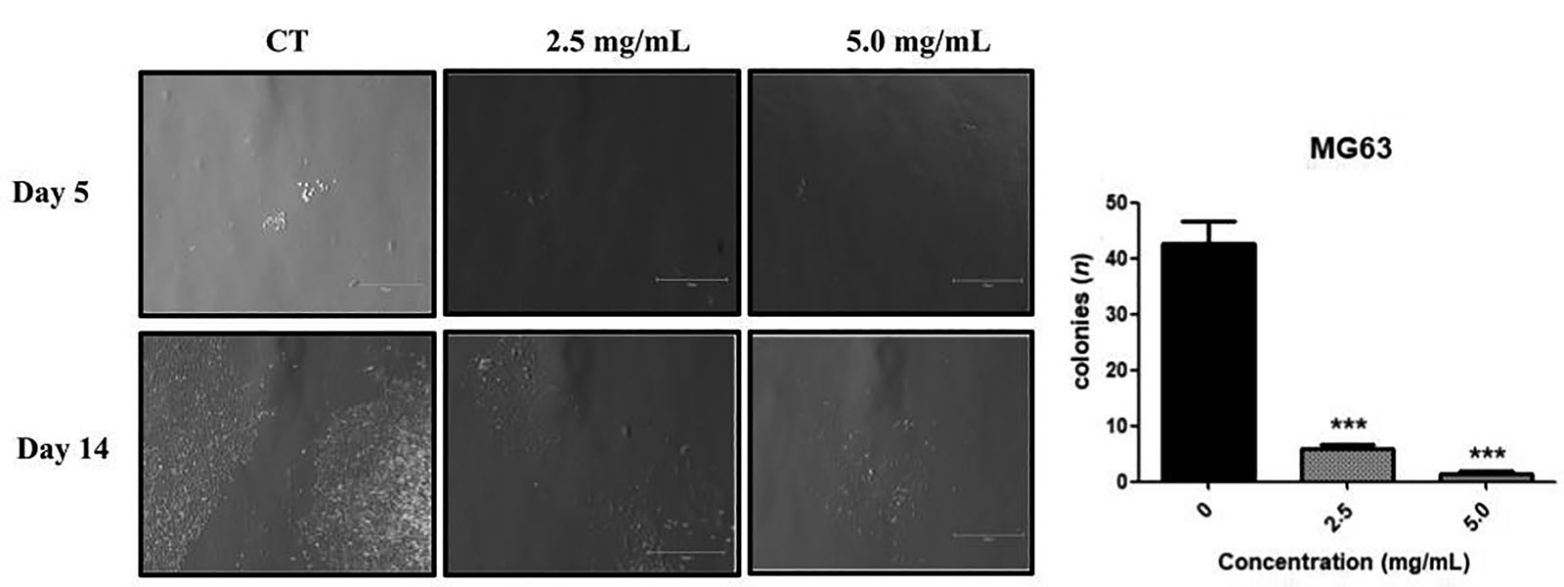
Colony formation assay with MG63 cell line under treatment with dehydrated mango pulp (PMD) at concentrations of 2.5 and 5 mg/mL, compared with no treatment. Representative images are shown on the left, and quantitative analysis of the total number of colonies formed after 14 days is presented on the right . The concentrations of 5 and 2.5 mg/mL were able to significantly inhibit colony formation when compared to the control group. One‐way ANOVA test with multiple comparisons of the Tukey test compared to the respective control group (^***^ *p* < 0.001). Scale bars = 750 µm.

The clonogenic or colony formation assay evaluates the ability of single cells to proliferate and form colonies of at least 50 viable cells. Inhibition of colony formation is of particular importance as clonogenic potential is closely associated with tumor progression and the ability to metastasize, key factors related to cancer recurrence and metastasis (Rafehi et al. 2011). In the clonogenic assay, DMP significantly reduced clonogenic potential in both MG63 and Caco‐2 cell lines. In MG63 cells, concentrations of 2.5 and 5.0 mg/mL markedly inhibited colony formation (*p* < 0.001) (Figure 2). In Caco‐2 cells, a significant reduction was observed only at 5.0 mg/mL (*p* < 0.05) (Figure 3). These findings are consistent with the decreased cell viability observed in the MTT assay for both lines.

### 3.5 DMP Increases Cell Apoptosis in Caco‐2 and MG63 Cell Lines

To investigate the mechanisms underlying the observed reduction in cell viability and inhibition of colony formation in MG63 and Caco‐2 cells treated with DMP, an apoptosis assay was performed using Annexin V and PI markers. Apoptosis is a key pathway associated with tumor suppression. The assay utilized the two highest doses tested in the MTT assay (2.5 and 5.0 mg/mL), consistent with the analysis approach used in the clonogenic assay.

**FIGURE 4:**
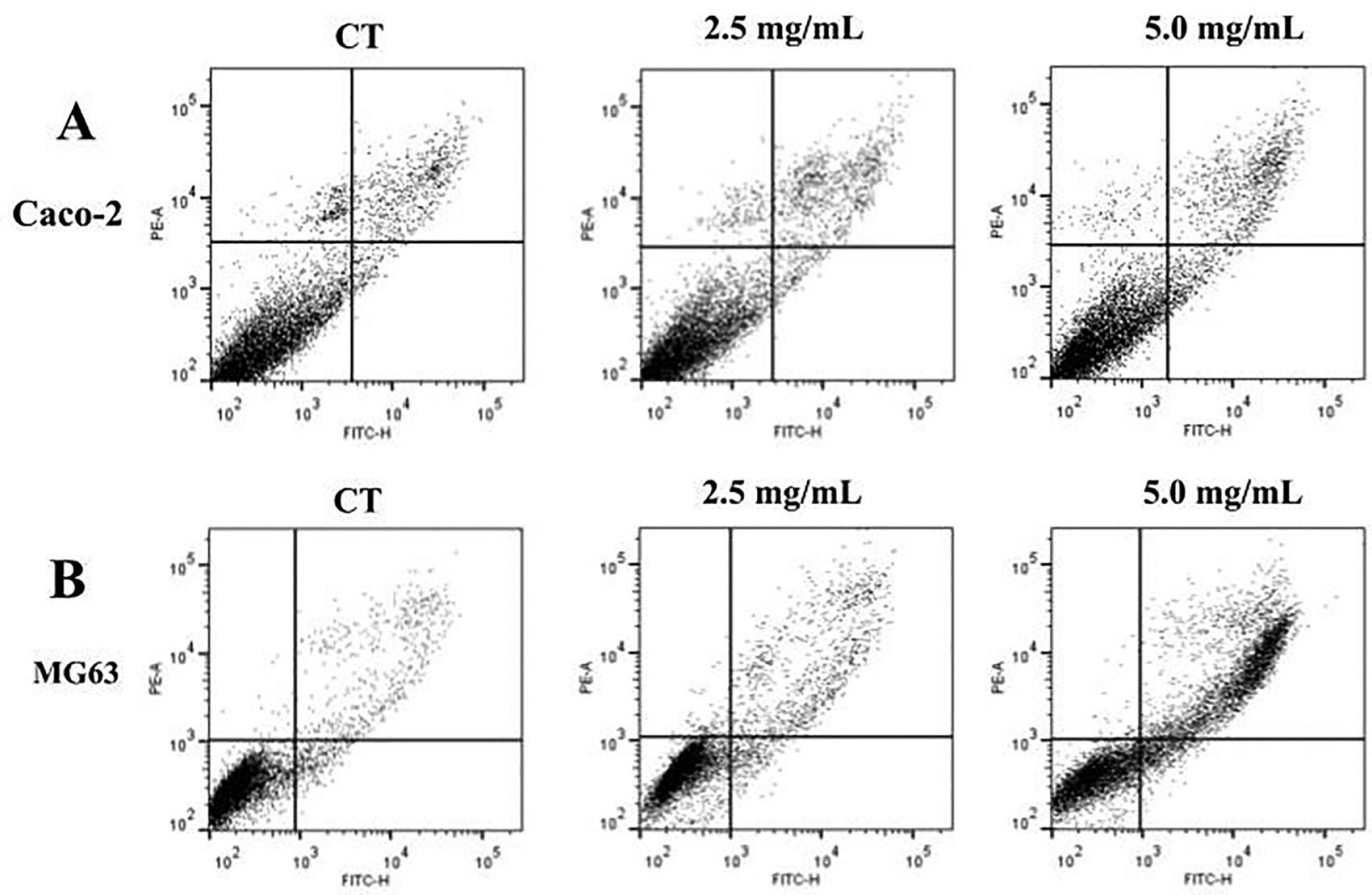
Dot plot graphs of Caco‐2 and MG63 cells with Annexin V‐FITC and PI markers.

It was observed that DMP significantly increased the initial apoptosis rates in Caco‐2 cells when compared to the control at a concentration of 5 mg/mL, as detailed in Table 6 and Figure 4. In MG63 cells, DMP exhibited a more pronounced pro‐apoptotic effect, with significant increases in both early and late apoptosis as well as in necrotic cell counts (*p* < 0.001), as shown in Table 7 and Figure 4. Furthermore, a 35.5% reduction in viable cell numbers was observed at the highest concentration of 5 mg/mL. These findings indicate that the observed reductions in cell viability in both Caco‐2 and MG63 cells are likely attributable to the induction of apoptosis.

**TABLE 6:** Apoptosis rate of Caco‐2 cell line treated with dried mango pulp (2, 5, and 5.0 mg/mL).

Treatment	Concentration	Viable cells	Initial apoptosis	Late apoptosis	Necrosis
Control	—	88.47 ± 0.76	1.7 ± 0.47	6.83 ± 0.73	3.01 ± 0.20
DMP	2.5 mg/mL	88.47 ± 1.32	1.81 ± 0.41	7.58 ± 0.54	2.14 ± 0.96
DMP	5.0 mg/mL	85.47 ± 3.60	3.28 ± 0.47^*^	8.43 ± 1.48	2.82 ± 2.77

*Note*: The concentration of 5 mg/mL was able to significantly increase the number of Caco‐2 cells in initial apoptosis when compared to the control (^*^ *p* < 0.01). Results are expressed as a percentage of total cells. One‐way ANOVA test with multiple comparisons of the Tukey test compared to the respective control group.

**TABLE 7:** Apoptosis rate of MG63 cell line treated with dried mango pulp (2, 5, and 5.0 mg/mL).

Treatment	Concentration	Viable cells	Initial apoptosis	Late apoptosis	Necrosis
Control	—	92.80 ± 0.01	2.55 ± 0.01	4.44 ± 0.01	0.21 ± 0.01
PMD	2,5 mg/mL	81.00 ± 6.92	2.99 ± 0.32	13.92 ± 7.70	2.09 ± 1.64
PMD	5,0 mg/mL	59.83 ± 4.58^*^	7.80 ± 0.46^*^	31.93 ± 4.86^*^	0.43 ± 0.12^*^

*Note*: The concentration of 5 mg/mL was able to significantly increase the number of MG63 cell lines in initial and late apoptosis when compared to the control (^*^ *p* < 0.001). In addition to significantly reducing the number of viable cells (^*^ *p* < 0.001). Results are expressed as a percentage of total cells. One‐way ANOVA test with multiple comparisons of the Tukey test compared to the respective control group.

The apoptotic effects observed in vitro (Tables 6 and 7) is consistent with previous studies on freeze‐dried mango extracts in colon cancer cells. Lozano‐Casabianca et al. (2022) demonstrated a significant reduction in cell viability of SW480 and SW620 cell lines after 48 h of exposure to an aqueous extract of freeze‐dried mango pulp at concentrations starting from 10 mg/mL. This effect was associated with cell cycle arrest at the G2/M phase, increased apoptosis, and elevated intracellular levels of reactive oxygen species (ROS). Furthermore, these extracts were shown to inhibit autophagy, a cellular process implicated in tumor progression (Lozano‐Casabianca et al. 2022, 2023; Vitto et al. 2022).

Although the biological assays were conducted using the whole DMP extract, the major phenolic compounds and carotenoids identified by HPLC are likely contributors to the observed biological effects. Previous studies have demonstrated that β‐carotene and chlorogenic acid exert cytotoxic and antiproliferative activities in cancer models, particularly in colorectal cancer. β‐Carotene has been reported to inhibit the self‐renewal capacity of human colon cancer cell lines (HCT116 and HT‐29), reduce tumor number and size, and delay tumor onset in xenograft mouse models, potentially through suppression of the Wnt/β‐catenin cancer stem cell (CSC) signaling pathway (Lee et al. 2022). Similarly, chlorogenic acid has shown marked antiproliferative activity in colorectal cancer models, including Caco‐2 cells, promoting S‐phase cell cycle arrest and preventing progression to the G2 phase (Cattivelli et al. 2023). Therefore, the relative abundance of these compounds in DMP suggests a plausible contribution to the antioxidant and antitumor responses observed in the present study.

Importantly, synergistic interactions among phytochemicals within the food matrix may further enhance biological activity (Chen et al. 2022), reinforcing the relevance of evaluating the whole product rather than isolated compounds.

To date, no studies have specifically investigated the antitumoral potential of mango extracts in MG63 osteosarcoma cells. However, related evidence supports the biological plausibility of such effects. Mangiferin, a bioactive compound identified in DMP, has demonstrated significant cytotoxic activity in osteosarcoma cell lines (SaOS‐2 and U2OS), reducing cell viability at 100 µM after 72 h (*p* < 0.01). These effects were associated with decreased cellular adhesion, migration, and invasion—key processes involved in tumor progression (Wen et al. 2020). In addition, in colorectal cancer models, mangiferin has been reported to induce apoptosis through modulation of apoptosis‐related signaling pathways (Li et al. 2016), further supporting its potential role in mediating antitumoral activity.

Furthermore, a carotenoid‐enriched nanoemulsion containing β‐carotene (49.2 µg/100 g), a compound also detected in DMP, was reported to induce cell death, enhance cytostatic autophagy, and promote cellular senescence in radiation‐resistant human osteosarcoma cells (SAOS400) at concentrations ranging from 100 to 200 µg/mL (Russo et al. 2022). Together, these findings support the hypothesis that mango‐derived bioactive compounds may exert antitumoral effects in osteosarcoma models.

In addition to their well‐known antioxidant effect—which protects against DNA damage and the development of cancer—phenolic compounds can also have antitumor effects by acting as pro‐oxidants under certain conditions. In cancer cells, these compounds can interact with elevated concentrations of redox‐active transition metals such as copper and iron and trigger Haber–Weiss and Fenton reactions. These reactions generate ROS that induce DNA cleavage and promote apoptosis (Slika et al. 2022). As a rich source of phenols, the pro‐oxidant activity of DMP could therefore play an important role in reducing the viability of cancer cells.

Taken together, our findings suggest that DMP extracts possess significant anticancer activity against colorectal adenocarcinoma and osteosarcoma cells, potentially via apoptosis induction. However, a major challenge in employing food matrices as adjuvants in non‐conventional therapies lies in ensuring the bioavailability and bioaccessibility of bioactive compounds, especially when targeting tissues such as bone in MG63 cells. Despite the complexities involved in absorption and systemic distribution, various bioactive compounds have been detected in bone and adjacent adipose tissues, indicating these sites as viable targets (Alldritt et al. 2019; Ermakov et al. 2013). Nevertheless, further in vivo studies and advanced in vitro models are essential to comprehensively evaluate DMP's bioavailability, therapeutic efficacy, and safety. Such investigations should also focus on defining effective dosage regimens that could meaningfully impact cancer prognosis. In addition, the molecular mechanisms underlying the apoptosis‐associated effects observed in this study were not directly examined. Future research incorporating the evaluation of canonical apoptotic signaling markers will be essential to confirm and further clarify the pathways involved.

From an applied perspective, identifying the predominant bioactive compounds in foam‐mat DMP may support breeding strategies aimed at enhancing phenolic and carotenoid content. Moreover, understanding how processing technologies influence phytochemical preservation may assist growers and processors in optimizing post‐harvest handling and drying conditions to minimize bioactive losses. These findings may also inform consumers regarding the functional potential of processed mango products.

## 4 Conclusion

DMP exhibited significant antioxidant potential and served as a valuable source of bioactive compounds, including carotenoids and phenolic compounds. In addition, it demonstrated in vitro antitumoral activity against colorectal adenocarcinoma (Caco‐2) and human osteosarcoma (MG63) cells, which was associated with increased apoptosis and reduced colony formation. However, further research is required to clarify the mechanisms underlying these effects and to evaluate their efficacy in vivo.

## Author Contributions

**Lia Igel Sodré**: conceptualization, writing – original draft, writing – review and editing, methodology, visualization, investigation, validation, software, formal analysis. **Rosana Bizon Carias**: conceptualization, investigation, methodology, supervision. **Priscila Borchio**: investigation, methodology, validation, visualization. **Pedro Paulo Saldanha Coimbra**: methodology, software, formal analysis, data curation, resources. **Ananda da Silva Antonio**: methodology, software, data curation, formal analysis, resources. **Henrique Marcelo Gualberto Pereira**: resources, methodology. **Maria Eduarda Flores**: methodology, investigation, software, data curation. **Carlos Eduardo Faria**: methodology, software, formal analysis. **Jorge da Silva Pinho**: methodology, writing – original draft, validation, data curation. **Vanessa Naciuk Castelo‐Branco**: methodology, validation, formal analysis, data curation. **Francine Albernaz Lobo**: methodology, validation, supervision, visualization, writing – original draft. **Valdir Florêncio da Veiga‐Junior**: software, formal analysis, resources, supervision. **Anderson Junger Teodoro**: resources, supervision, data curation, visualization, validation, writing – review and editing, project administration.

## Conflicts of Interest

The authors declare no conflicts of interest.

## Supporting information



A—Chromatographic profile of Dried Mango Pulp—HPLC
B—Certificate of approval by the Research Ethics Committee
C—Complete analysis of metabolomic profile of Dried Mango Pulp
