# Supplementary material for: Antioxidant Activity, Phytochemical Characterization, and In Vitro Antitumor Effects of Foam‐Mat Dried Mango (Mangifera indica L.) Pulp
Source: J Food Sci. 2026 Apr 14;91:e71024. doi: 10.1111/1750-3841.71024 (PMC13080074; doi:10.1111/1750-3841.71024)
Supplement: Supplementary file 1 — A—Chromatographic profile of Dried Mango Pulp—HPLC B—Certificate of approval by the Research Ethics Committee C—Complete analysis of metabolomic profile of Dried Mango Pulp [file JFDS-91-0-s001.docx]

**SUPPLEMENTARY MATERIAL – S1**

**A - Chromatographic profile of Dried Mango Pulp - HPLC**

**Figure A:** Chromatographic profile of carotenoid and phenolic compounds identified in dehydrated mango pulp. Quantification was performed by external calibration from the areas of the chromatographic peaks obtained by DAD detection at 450 nm for carotenoid compounds, and 254, 310, 320 and 325 nm for phenolic compounds.


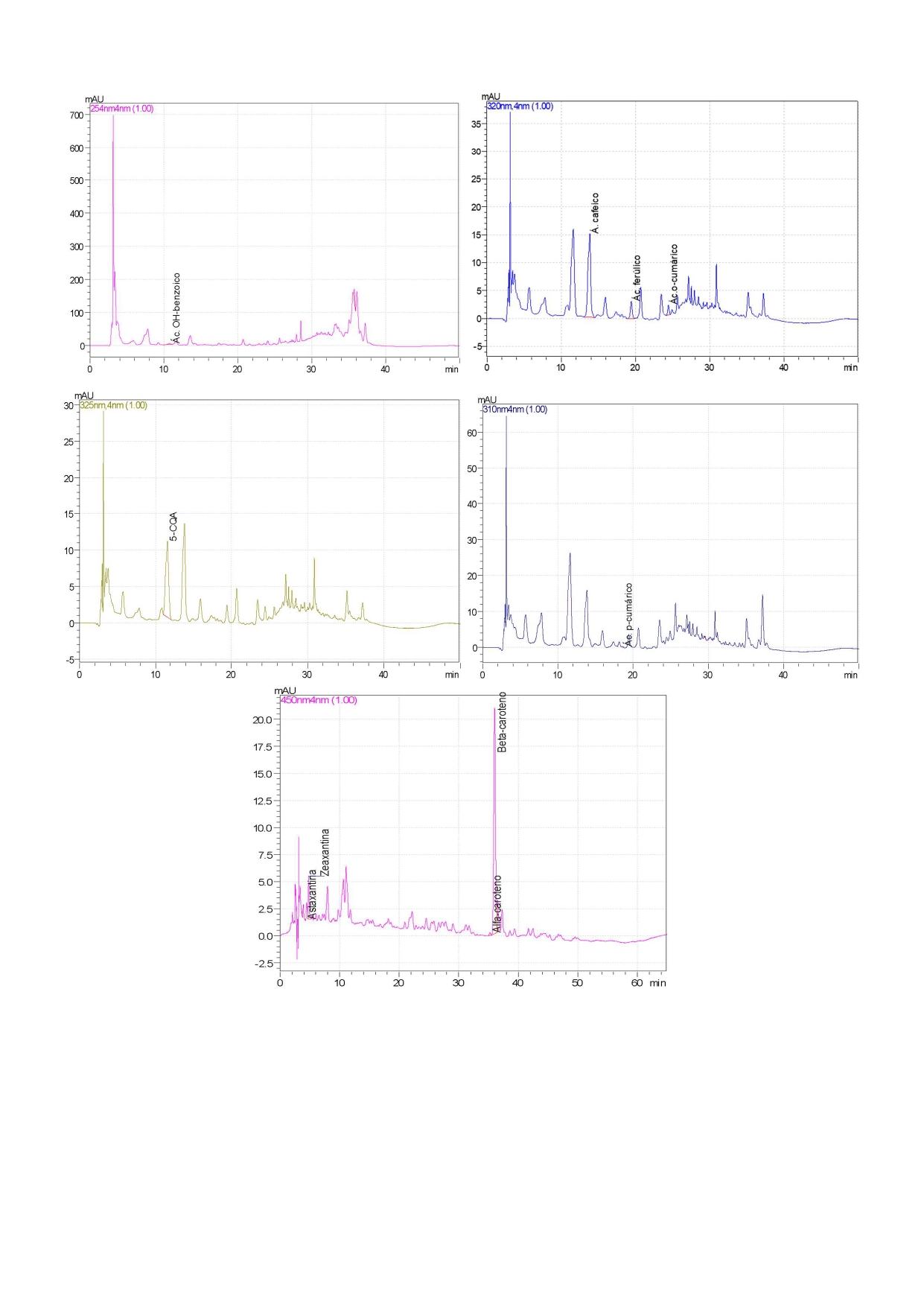


**B -** **Certificate of approval by the Research Ethics Committe**


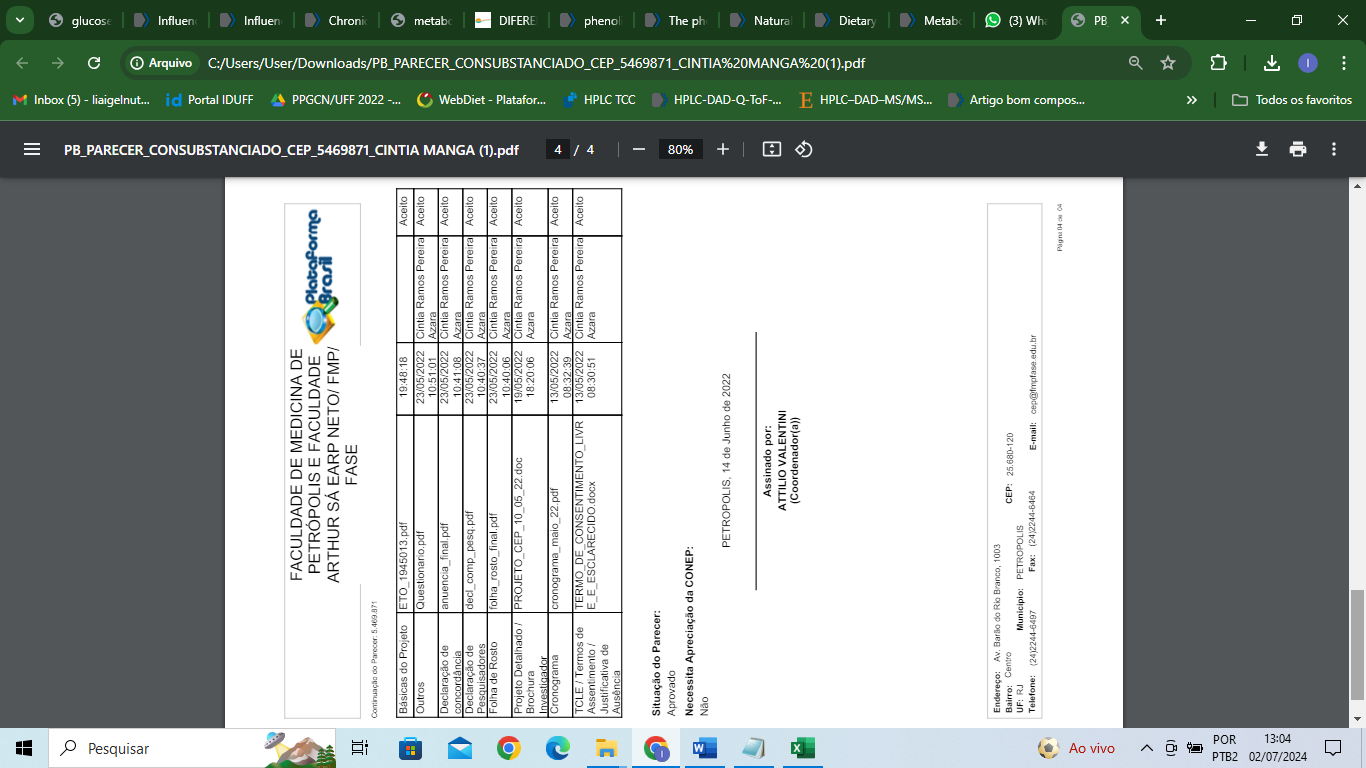


**C - Complete Analysis of metabolomic profile os Dried Mango Pulp**

**Table .** Identified compounds in dried mango pulp extract

|  | **Compound** | **Molecular formula** | **Adduct** | **Theorical m/z** | **Observed m/z** | **Error (ppm)** | **Peak Area** | **Ref.** |
| --- | --- | --- | --- | --- | --- | --- | --- | --- |
| **1** | Mangiferin | C19H18O11 | [M - H]-/[M +H]+ | 421.0776 | 421.0779 | 0.71 | 6400000 | Ybañez-Julca, 2020 |
| **2** | Naringenin-7-O-glucoside | C21H22O10 | [M - H]**^+^** | 435.129 | 435.1291 | -8.04 | 5100000 | GNPS |
| **3** | Gallic acid 4-O-glucoside | C13H16O10 | [M - H]**^-^** | 331.067 | 331.0672 | 0.06 | 520000 | GNPS |
| **4** | Shikimic acid | C7H10O5 | [M - H]^-^ | 173.05 | 173.0445 | 5.00 | 3200000 | GNPS |
| **5** | Linolenic acid | C18H30O2 | [M - H]**^+^** | 279.232 | 279.2317 | -1,07 | 15000000 | GNPS |
| **6** | Trihydroxyoctadienoic acid (Trihydroxylinoleic acid) | C18H_31_O_5_ | [M - H]^-^ | 327.2177 | 327.2177 | 0 | 710000 | Ybañez-Julca, 2020 |
| **7** | Trihydroxyoctaenoic acid | C18H33O5 | [M - H]^-^ | 329.2333 | 329.2333 | -0.15 | 15000000 | LEE, 2021 |
| **8** | 3-Feruloylquinic acid | C_17_H_20_O_9_ | [M - H]- | 367.1034 | 367.1047 | 3.54 | 3800000 | LEE, 2022 |
| **9** | Citric acid | C6H8O7 | [M - H]^-^ | 191.02 | 191.0188 | -6.28 | 22000000 | GNPS |
| **10** | Octadecenoic acid | C18H34O2 | [M - H]^-^ | 295.228 | 295.2276 | -1.35 | 7600000 | GNPS |
| **11** | Quinic acid | C_7_H_12_O_6_ | [M - H]^-^ | 191.0561 | 191.0551 | -5,286405 | 18000000 | Ybañez-Julca, 2020 |
| **12** | Hexadecanoyl-hydroxy-sn-glycerophosphocholine | [C_25_H_52_NO_9_P](http://www.chemspider.com/Search.aspx?q=C25H52NO9P) | [M - H]^-^ | 540.326 | 540.3306 | 8.51 | 1400000 | GNPS |
| **13** | 1-(9Z,12Z-octadecadienoyl)-sn-glycero-3 phosphocholine | C26H50NO7P | [M - H]^-^ | 564.329 | 564.3304 | 2,48 | 4100000 | GNPS |
| **14** | Fructose | C₆H₁₂O₆ | [M - H]^-^ | 179.056 | 179.0551 | -5.02 | 31000000 | GNPS |
| **15** | Lysolecithins | C26H52NO7P | [M + H]**^+^** | 496.339 | 496.339 | 0 | 14000000 | GNPS |
| **16** | Sucrose | C₁₂H₂₂O₁₁ | [M - H]^-^ | 341.109 | 341.1086 | 1.17 | 150000000 | GNPS |
| **17** | Alpha-D-Glucopyranose | C6H12O | [M - H]^-^ | 225.062 | 225.061 | -4.44 | 180000000 | GNPS |
| **18** | (9S)-Hydroxyoctadecadienoic acid | [C_18_H_32_O_3_](http://www.chemspider.com/Search.aspx?q=C18H32O3) | [M - H]^-^ | 295.228 | 295.2276 | 1.35 | 7600000 | GNPS |
| **19** | 1-Oleoyl-L-.alpha.-lysophosphatidic acid | C21H41O7P | [M - H]^-^ | 435.252 | 435.2515 | -1.14 | 1500000 | GNPS |
| **20** | Alpha-Trehalose | C12H22O11 | [M - H]^-^ | 387.115 | 387.1142 | 2.06 | 360000000 | GNPS |
| **21** | 1-Palmitoyl-2-hydroxy-sn-glycero-3-phosphoethanolamine | [C_21_H_44_NO_7_P](https://pubchem.ncbi.nlm.nih.gov/#query%3DC21H44NO7P) | [M - H]^-^ | 452.279 | 452.2781 | -1.98 | 1200000 | GNPS |
| **22** | Palatinose | [C_12_H_22_O_11_](https://pubchem.ncbi.nlm.nih.gov/#query%3DC12H22O11) | [2M - H]^-^ | 683.225 | 683.2249 | 0.14 | 24000000 | GNPS |
